# Supplementary material for: MetaRibo-Seq measures translation in microbiomes
Source: Nat Commun. 2020 Jun 29;11:3268. doi: 10.1038/s41467-020-17081-z (PMC7324362; doi:10.1038/s41467-020-17081-z)
Supplement: Supplementary file 10 — Supplementary Data 7 [file 41467_2020_17081_MOESM10_ESM.zip › File2/Confidence_High_Taxonomy/194812_out.krona.html]

Javascript must be enabled to view this page.

members
magnitude
magnitudeUnassigned
count
unassigned
taxon
rank

194812\_out

3

superkingdom
2
3

phylum
3
1239

186801
3
class

3
186802
order

3
541000
family

genus
1263
3

species

SRS015217\_contig\_number\_contig-100\_1136.155903
1
2293228

1
2293160

SRS050422\_contig\_number\_34998
species


SRS1055083\_contig\_number\_11529
1
2293209
species
